# Analysis of myocardial work in different obesity phenotypes and its association with epicardial adipose tissue

**DOI:** 10.1186/s12872-026-06298-3

**Published:** 2026-07-22

**Authors:** Zhenjiang Wang, Chaodi Tan, Zhuoxin Lyu, Zhuzhi Wen, Tao Wu, Dengfeng Geng, Shaoxin Zheng

**Affiliations:** 1 Department of Cardiology, Sun Yat-sen Memorial Hospital, Sun Yat-sen University, Guangzhou, Guangdong 510145 China; 2 Department of Cardiology, Zhongshan Hospital, Fudan University, Shanghai, China

**Keywords:** Obesity phenotype, Metabolic syndrome, Myocardial work, Subclinical cardiac dysfunction, Epicardial adipose tissue

## Abstract

**Objective:**

To evaluate subclinical cardiac dysfunction across distinct obesity phenotypes using myocardial work (MW) indices, and to investigate the moderating role of epicardial adipose tissue (EAT) on MW impairment.

**Methods:**

One hundred ninety-two participants were categorized by BMI and metabolic status into four groups: metabolically healthy normal weight (MHNW), metabolically healthy obesity (MHO), metabolically unhealthy normal weight (MUNW), and metabolically unhealthy obesity (MUO). MW indices were measured using speckle-tracking echocardiography. EAT volume was quantified from ECG-gated CT scans. Associations were analyzed using ANCOVA and regression models.

**Results:**

Despite no significant differences in left ventricular ejection fraction (LVEF) among the groups, MUNW and MUO individuals exhibited significantly impaired MW, characterized by lower global work index (GWI), global constructive work (GCW), and global work efficiency (GWE), alongside higher global waste work (GWW), compared with MHNW controls. MHO patients showed significantly better MW profiles than MUNW and MUO groups. EAT volume was increased in all obesity phenotype groups. Although no direct association was found between EAT volume and MW indices, EAT volume significantly moderated the relationship between Metabolically Unhealthy (MUH) and both GWE and GWW.

**Conclusions:**

MW detects subclinical dysfunction earlier than LVEF across obesity phenotypes, with impairment progressively worsening from MHO to MUNW to MUO. In metabolically unhealthy individuals with increased EAT volume, more pronounced reductions in GWE and elevations in GWW were observed.

**Trial Rrgistration:**

This study has been registered in the Chinese Clinical Trial Registry under the registration number *ChiCTR2500100754* (registration date: April 15, 2025).

**Supplementary Information:**

The online version contains supplementary material available at https://doi.org/10.1186/s12872-026-06298-3.

## Introduction

Obesity is defined as excessive or abnormal fat accumulation that may impair health. Multiple obesity-related mechanisms, including endocrine disturbances, alterations in cardiac structure and function, and hemodynamic changes, are considered fundamental contributors to atherosclerosis and cardiac dysfunction [1]. Obesity itself is also recognized as an independent cardiometabolic risk factor and is strongly associated with heart failure with preserved ejection fraction (HFpEF) [2].

Nevertheless, obesity presents with considerable heterogeneity across individuals. Assessing obesity solely by body mass index (BMI) is insufficient for individualized prediction of cardiometabolic risk. Sims et al. [3] reported that some individuals with elevated BMI exhibited lower long-term cardiovascular event rates and mortality. Conversely, individuals with normal BMI but with obesity-related metabolic syndrome (MetS) displayed substantially increased cardiometabolic risk [4]. The concept of “obesity phenotypes”, which integrates BMI and metabolic health status, enables classification of obesity into distinct subtypes, thereby capturing its heterogeneity and improving prediction of cardiovascular risk.

Within this framework, BMI, metabolic health, and abnormal fat accumulation are critical determinants of cardiovascular outcomes. Impaired expansion of subcutaneous adipose tissue promotes excessive abdominal deposition, leading to visceral fat, which can further accumulate ectopically in the liver and around the heart. These ectopic fat depots secrete large amounts of inflammatory mediators that may injure surrounding organs [5]. Epicardial adipose tissue (EAT), located around the heart without a fascial layer separating it from the myocardium [6], is capable of direct interaction with myocardial tissue. Under physiological conditions, EAT provides cushioning and protection to the myocardium [7, 8]. In contrast, under pathological conditions, chronic low-grade inflammation induces secretion of proinflammatory and profibrotic cytokines, contributing to myocardial remodeling and dysfunction [8, 9]. Lipid infiltration within the myocardium has been associated with fibrosis, apoptosis, and contractile abnormalities, ultimately leading to HFpEF and related complications [10, 11]. Prior studies have suggested that individuals with higher BMI are at increased risk of subclinical left ventricular (LV) systolic dysfunction [12], yet the extent of subclinical LV impairment across different obesity phenotypes remains unclear.

Currently, left ventricular ejection fraction (LVEF) is widely used in clinical practice. However, it lacks sensitivity for detecting early cardiac dysfunction in stage B heart failure (preclinical phase), when structural and overt functional changes have not yet occurred. Although LVGLS is a sensitive marker of subclinical myocardial dysfunction, its abnormalities are influenced by both intrinsic myocardial contractility and left ventricular afterload. Given that obesity and metabolic disorders are frequently accompanied by elevated blood pressure and altered loading conditions, interpretation of LVGLS alone may be challenging. To address these limitations, Russell et al. [13] developed a novel method based on two-dimensional speckle-tracking echocardiography that integrates noninvasive brachial cuff pressure with LV global longitudinal strain (LVGLS). By combining strain analysis with valve timing events, a noninvasive pressure–strain loop (PSL) can be generated to quantify global myocardial work (MW). This method reduces load dependency compared with LVEF and GLS alone, and has been validated for the detection of subclinical LV dysfunction in various cardiovascular diseases [14, 15].

As a marker of abnormal fat distribution, EAT volume may have important implications for cardiac function in individuals with obesity. Arnold et al. [16] demonstrated that increased EAT volume index is independently associated with insulin resistance, myocardial fibrosis burden, and impaired LVGLS. However, the utility of MW in detecting subclinical cardiac dysfunction across different obesity phenotypes has not yet been investigated. In addition, the relationship between EAT volume and MW remains unclear. We hypothesized that myocardial work impairment differs across obesity phenotypes and is more pronounced in metabolically unhealthy individuals. We also hypothesized that EAT volume may influence the relationship between metabolic status and myocardial work. Therefore, this study aimed to evaluate myocardial work impairment across different obesity phenotypes and to investigate the potential role of EAT volume in myocardial work alterations.

## Method

### Study design and participants

**Fig. 1:**
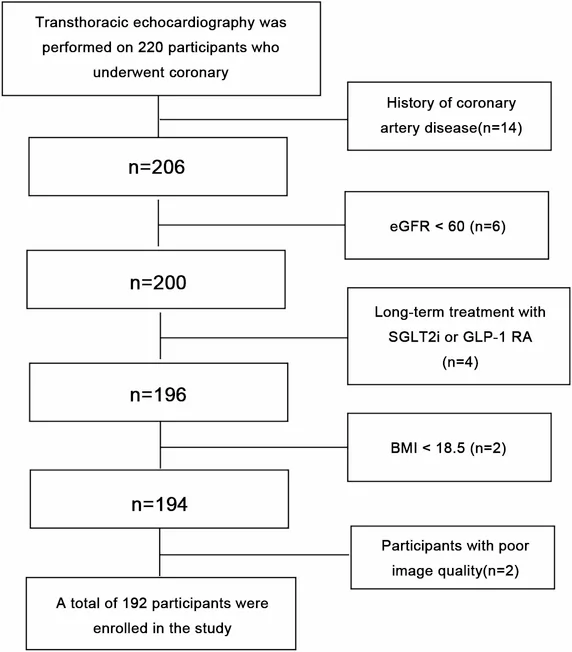
Inclusion and Exclusion of Cases

This was a single-center, observational, cross-sectional study. We evaluated consecutive adult patients (age > 18 years) who underwent coronary computed tomography angiography (CTA) for suspected coronary artery disease at the Department of Cardiology, Sun Yat-sen Memorial Hospital, between May 2024 and February 2025. Only patients with no clinical or echocardiographic evidence of cardiac dysfunction were ultimately included in this analysis. Patients initially presented for coronary CTA due to symptoms such as chest tightness or for screening of suspected coronary artery disease. However, to minimize confounding factors, individuals with confirmed coronary artery disease (e.g., luminal stenosis ≥ 50%) on the current CTA or a prior history of coronary artery disease were strictly excluded. Patients with any clinical condition that could cause a discrepancy between noninvasive brachial systolic blood pressure and left ventricular systolic pressure were excluded. Additional exclusion criteria included cardiomyopathy, valvular heart disease, arrhythmias, high-output conditions, chronic kidney disease, or malignancy. Patients with long-term (≥ 6 months) use of medications affecting body weight and epicardial adipose tissue (EAT) volume, such as sodium-glucose cotransporter 2 inhibitors (SGLT-2i) or glucagon-like peptide-1 receptor agonists (GLP-1 RA), were also excluded. Individuals with extremely low (BMI < 18.5 kg/m²) or extremely high (BMI > 40 kg/m²) body weight, incomplete data, or poor-quality imaging that precluded analysis were excluded. Furthermore, this study was registered after the completion of data collection due to its observational and non-interventional design. Nevertheless, all study protocols, inclusion and exclusion criteria, and target endpoints were established a priori and strictly adhered to without any modifications. Based on these criteria, a total of 192 participants were included in the study, and the screening flowchart is shown in Fig. 1.

Demographic data were recorded, and BMI, body surface area (BSA), and waist-to-hip ratio (WHR) were calculated. Systolic and diastolic blood pressure were measured immediately before echocardiography. Laboratory tests obtained at admission included fasting plasma glucose (FPG), glycated hemoglobin (HbA1c), NT-proBNP, estimated glomerular filtration rate (eGFR) and blood lipids.

To ensure optimal heart rate control and hemodynamic stability for contrast-enhanced coronary CTA, all participants were strictly instructed to completely abstain from smoking, alcohol, and caffeine-containing beverages for at least 12 h prior to the imaging and clinical examinations.

### Definition of obesity phenotypes

Given that Asian populations may exhibit obesity-related disease risk at relatively lower BMI levels, we adopted the World Health Organization (WHO) BMI classification and the WHO Western Pacific Regional recommendations [17]. Individuals with BMI ≥ 25.0 kg/m² were considered to have elevated cardiometabolic risk and were uniformly classified as “overweight/obese” [17], while those with 18.5 ≤ BMI < 25.0 kg/m² were defined as “normal weight.”

MetS was defined according to the revised 2005 National Cholesterol Education Program Adult Treatment Panel III criteria [18], requiring the presence of three or more of the following conditions:

- ① Waist circumference (WC) ≥ 90 cm in men or ≥ 80 cm in women;
- ② FPG ≥ 5.6 mmol/L, a self-reported history of diabetes, or current use of glucose-lowering medications;
- ③ Systolic blood pressure ≥ 130 mmHg and/or diastolic blood pressure ≥ 85 mmHg, a self-reported history of hypertension (HBP), or current use of antihypertensive medications;
- ④ High-density lipoprotein cholesterol (HDL-C) < 1.0 mmol/L in men or < 1.3 mmol/L in women;
- ⑤ Triglycerides (TG) ≥ 1.7 mmol/L or current use of lipid-lowering medications.

Based on these criteria, participants were explicitly defined as ‘Metabolically Unhealthy (MUH)’ if they met three or more of the conditions (i.e., presence of MetS). Conversely, participants meeting two or fewer conditions were defined as ‘Metabolically Healthy (MH).

Combining BMI categories (normal weight or overweight/obese) with metabolic health status (presence or absence of MetS), participants were classified into four groups: Metabolically healthy normal weight (MHNW, control group), Metabolically healthy overweight/obese (MHO), Metabolically unhealthy normal weight (MUNW) and Metabolically unhealthy overweight/obese (MUO).

A point to emphasize regarding the differences in baseline comorbidities and medication use across different obesity phenotypes is that, according to the NCEP ATP III guidelines, pharmacological treatment for elevated blood pressure, hyperglycemia, and other such conditions, as well as the comorbidities themselves, are explicit diagnostic criteria for the metabolically unhealthy (MUH) status. Therefore, forcibly adjusting for these binary medication variables in statistical analysis would introduce severe statistical multicollinearity and over-adjustment.

### Echocardiographic examination

Echocardiographic examinations were performed by experienced cardiac sonographers using a GE Vivid E9 ultrasound system equipped with an M5s 3.5–5 MHz transducer. All standard and speckle-tracking echocardiographic images (three consecutive cardiac cycles) were stored digitally for offline analysis. To minimize measurement bias and ensure rigorous quality control, the offline image reading, myocardial strain contouring, and MW analyses were performed independently by two experienced echocardiographers who were strictly blinded to the participants’ clinical characteristics, metabolic grouping, and CT imaging results. Global left ventricular longitudinal strain (LVGLS) and left atrial strain (LAS) were measured using EchoPAC software (version 204). Based on strain data and non-invasively measured systolic blood pressure, left ventricular pressure–strain loops were constructed to derive global myocardial work indices. The software automatically calculated the following parameters: (1) GWI (Global Work Index), defined as the average work performed by the left ventricular myocardium from mitral valve closure to mitral valve opening; (2) GCW (Global Constructive Work), representing the work performed by left ventricular myocardial shortening during systole and lengthening during isovolumic relaxation; (3) GWW (Global Waste Work), representing the work performed by left ventricular myocardial lengthening during systole and shortening during isovolumic relaxation; and (4) GWE (Global Work Efficiency), calculated as GCW / (GCW + GWW) × 100% [12].

### Computed Tomography (CT) imaging

All imaging data were acquired using a 128-slice CT scanner (Siemens Somatom Flash, Erlangen, Germany). Contrast-enhanced ECG-gated coronary CT angiography was performed with prospective ECG triggering at 70% of the R–R interval and a tube voltage of 120 kVp. Raw data were reconstructed at a slice thickness of 3.0 mm for EAT assessment. The reconstructed images were transferred to a Siemens Syngo.Via workstation, where EAT was delineated by two experienced cardiovascular radiologists. A fat attenuation threshold of − 190 to − 30 HU was applied. The software automatically identified the pulmonary artery bifurcation and the posterior descending artery as the upper and lower anatomical boundaries, respectively, segmented the EAT region, and calculated the EAT volume [19]. The EAT measurements demonstrated good intra- and inter-observer reliability (ICC > 0.85).

### Statistical analysis

Normality of continuous variables was assessed using Q-Q plots. Continuous variables with a normal distribution were presented as mean ± SD, while non-normally distributed variables were expressed as median (IQR). Categorical variables were expressed as counts and percentages. Comparisons between two groups were performed using the independent t-test or Mann–Whitney U test for continuous variables and the chi-square test or Fisher’s exact test for categorical variables. ANCOVA with Tukey’s post hoc multiple comparison test was applied to compare differences in MW parameters and EAT volume between groups with obesity and control groups as well as among different obesity phenotypes.

Univariate and multivariate linear regression analyses were conducted to evaluate associations of MW parameters with metabolic syndrome, BMI, EAT volume, and other clinical variables. Interaction terms between Metabolically Unhealthy (MUH) and EAT volume were included in multivariable linear regression models to examine the moderating effect of EAT volume on myocardial work. Inter- and intra-observer variability of GWI, GCW, GWW, and GWE was assessed by randomly selecting 20 participants from both the group with obesity and normal-weight groups for repeated measurements. Analyses were performed using R 4.0.2. A two-sided *P* value < 0.05 was considered statistically significant.

## Results

### Baseline characteristics and laboratory findings of control and obesity phenotype groups (Table 1)

**Table 1:** Characteristics of Participants

	MHNW (*N* = 63)	MHO (*N* = 39)	MUNW (*N* = 35)	MUO (*N* = 55)	*p*-value
Male, *n* (%)	40 (63.5)	19 (48.7)	17 (48.6)	21 (38.2)	0.053
Age, year	58 ± 12	59 ± 11	64 ± 10	56 ± 11	0.011
Height, cm	161.1 ± 7.3	161.9 ± 6.7	163.1 ± 6.6	164.3 ± 7.8	0.101
Weight, kg	57.0 ± 6.8	70.7 ± 8.5	61.0 ± 7.4	75.9 ± 9.8	< 0.001
BMI, kg/m^2^	21.9 ± 1.6	26.9 ± 2.3	22.9 ± 1.7	28.0 ± 2.5	< 0.001
BSA, m^2^	1.6 ± 0.1	1.7 ± 0.1	1.6 ± 0.1	1.8 ± 0.2	< 0.001
WC, cm	79.6 ± 5.1	91.8 ± 9.5	89.9 ± 5.6	98.1 ± 7.1	< 0.001
WHR	0.88 ± 0.05	0.93 ± 0.07	0.97 ± 0.05	0.97 ± 0.06	< 0.001
SBP, mmHg	129 ± 18	129 ± 16	133 ± 18	139 ± 17	0.005
DBP, mmHg	79 ± 11	79 ± 12	81 ± 12	89 ± 13	< 0.001
HBP, *n* (%)	27 (42.9)	16 (41)	25 (71.4)	48 (87.3)	< 0.001
DM, *n* (%)	7 (11.1)	5 (12.8)	11 (31.4)	20 (36.4)	0.002
History of smoking					0.298
Never, *n* (%)	49 (77.8)	28 (71.8)	24 (68.6)	32 (58.2)	
quit, *n* (%)	4 (6.3%)	2 (5.1%)	3 (8.6%)	3 (5.5%)	
Current, *n* (%)	10 (15.9)	9 (23.1)	8 (22.9)	20 (36.4)	
Drinking, *n* (%)	59 (93.7)	36 (92.3)	32 (91.4)	49 (89.1)	0.843
Medication Usage					
Statins, *n* (%)	17 (27)	13 (33.3)	13 (37.1)	19 (34.5)	0.721
Antihypertensions, *n* (%)	21 (33.3)	14 (35.9)	24 (68.6)	41 (74.5)	< 0.001
Hypoglycemic drugs, *n* (%)	5 (7.9)	3 (7.7)	8 (22.9)	10 (18.2)	0.092
NT-proBNP, pg/dL	61.7 ± 51.8	48.9 ± 47.6	48.9 ± 47.3	31.3 ± 30.5	0.004
TC, mmol/L	5.0 ± 1.1	4.6 ± 1.2	5.0 ± 1.7	5.0 ± 1.1	0.414
HDL-C, mmol/L	1.33 ± 0.26	1.20 ± 0.21	1.11 ± 0.24	1.03 ± 0.22	< 0.001
LDL-C, mmol/L	3.15 ± 0.91	2.96 ± 0.94	3.14 ± 0.97	3.26 ± 0.78	0.463
TG, mmol/L	1.15 ± 0.46	1.22 ± 0.49	2.24 ± 1.76	2.22 ± 1.35	< 0.001
FPG, mmol/L	5.02 ± 0.72	5.04 ± 1.00	5.95 ± 2.00	6.18 ± 1.68	< 0.001
HbA1c	5.59 ± 0.67	5.83 ± 0.78	6.77 ± 2.04	6.40 ± 1.37	< 0.001
eGFR, mL/min/1.73m^2^	100.3 ± 9.7	93.1 ± 12.8	92.7 ± 14.1	96.4 ± 14.1	0.009

*Abbreviations*: *BMI* Body Mass Index, *BSA* Body Surface Area, *WC* Waist Circumference, *HC* Hip Circumference, *WHR* Waist-Hip Ratio, *SBP* Systolic Blood Pressure, *DBP* Diastolic Blood Pressure, *HBP* Hypertension, *DM* Diabetes Mellitus, *NT-proBNP* N-terminal pro–B-type Natriuretic Peptide, *TC* Total Cholesterol, *HDL-C* High-Density Lipoprotein Cholesterol, *LDL-C* Low-Density Lipoprotein Cholesterol, *TG* Triglycerides, *FPG* Fasting Plasma Glucose

A total of 192 participants were included: 63 in the MHNW group, 39 in the MHO group, 35 in the MUNW group, and 55 in the MUO group. Compared with the MHNW, the obesity phenotype groups showed significant differences in age, body weight, BMI, BSA and WHR (*P* < 0.05). Regarding laboratory parameters, TG, FPG, and HbA1c were significantly higher in obesity phenotype groups than in controls, while HDL-C and eGFR were significantly lower (*P* < 0.01).

### Conventional echocardiography and myocardial strain in control and obesity phenotype groups (Table 2)

**Table 2:** Transthoracic Echocardiography Data

	MHNW (*N* = 63)	MHO (*N* = 39)	MUNW (*N* = 35)	MUO (*N* = 55)	*p*-value
Conventional Echocardiographic Parameters					
LAd, mm	32.9 ± 2.8	35.3 ± 3.5	34.1 ± 3.2	36.1 ± 3.9	< 0.001
LAVmax, mL	30.3 ± 10.2	34.0 ± 11.4	29.4 ± 11.3	33.2 ± 12.6	0.183
LAVI, mL/m^2^	19.5 ± 6.5	19.8 ± 7.2	18.1 ± 7.1	18.3 ± 6.7	0.564
LVEDd, mm	45.8 ± 3.3	47.5 ± 3.6	47.5 ± 3.5	48.1 ± 3.3	0.002
LVPWd, mm	8.4 ± 1.1	9.1 ± 0.8	9.1 ± 1.3	9.7 ± 1.2	< 0.001
IVSd, mm	8.6 ± 1.3	9.3 ± 1.0	9.4 ± 1.4	10.1 ± 1.5	< 0.001
LVM, g	128.9 ± 30.8	150.2 ± 27.5	152.1 ± 33.4	169.7 ± 40.0	< 0.001
LVMI, g/m^2^	82.3 ± 16.9	86.3 ± 14.2	93.7 ± 19.2	75.6 ± 13.6	0.714
LVEF, %	67.8 ± 4.3	67.7 ± 4.2	67.4 ± 3.8	67.6 ± 4.7	0.979
E, m/s	72.3 ± 16.2	69.3 ± 15.4	64.7 ± 15.1	63.5 ± 14.0	0.010
A, m/s	79.7 ± 18.2	81.4 ± 18.1	88.3 ± 15.6	85.5 ± 15.5	0.066
E/A	1.0 ± 0.3	0.9 ± 0.3	0.7 ± 0.2	0.8 ± 0.2	< 0.001
e’, m/s	7.6 ± 2.2	6.9 ± 1.8	6.1 ± 1.7	5.8 ± 1.6	< 0.001
E/e’	10.0 ± 2.5	10.5 ± 2.5	11.1 ± 3.1	11.6 ± 3.3	0.032
Speckle-Tracking Echocardiography					
LVGLS, %	-19.5 ± 1.9	-19.1 ± 1.7	-16.8 ± 1.8	-15.5 ± 1.7	< 0.001
LASr, %	35.7 ± 9.1	33.7 ± 6.8	29.5 ± 7.1	31.2 ± 7.1	< 0.001
LAScd, %	-18.1 ± 7.7	-15.3 ± 6.1	-12.2 ± 3.9	-14.0 ± 5.7	< 0.001
LASct, %	-17.6 ± 6.3	-18.4 ± 5.3	-17.3 ± 5.3	-17.2 ± 5.3	0.763
BP					
SBP, mmHg	129 ± 18	129 ± 16	133 ± 18	139 ± 17	0.005
DBP, mmHg	79 ± 11	79 ± 12	81 ± 12	89 ± 13	< 0.001

*Abbreviations*: *LAd* Left Atrial Diameter, *LAVmax* Maximum Left Atrial Volume, *LAVI* Left Atrial Volume Index, *LVEDd* Left Ventricular End-Diastolic Diameter, *IVSd* Interventricular Septal Thickness, *LVPWd* Left Ventricular Posterior Wall Thickness, *LVEF* Left Ventricular Ejection Fraction, *E* Early Diastolic Mitral Inflow Velocity, *A* Late Diastolic Mitral Inflow Velocity, *e*’ Average Early Diastolic Mitral Annular Velocity (septal and lateral), *LVGLS* Left Ventricular Global Longitudinal Strain, *LASr* Left Atrial Reservoir Strain, *LAScd* Left Atrial Conduit Strain, *LASct* Left Atrial Contraction Strain, *SBP* Systolic Blood Pressure, *DBP* Diastolic Blood Pressure

LVPWd, IVSd, and LVM were significantly lower in the control group compared with obesity phenotype groups, whereas LVMI and LVEF did not differ significantly (*P* > 0.05). The absolute value of LVGLS in the control group was significantly higher than in the MUNW and MUO (*P* < 0.001), but did not differ from the MHO (*P* > 0.05). For all obesity phenotype groups, LASr and LAScd differed significantly from the control group (*P* < 0.05), whereas LASct showed no significant differences (*P* > 0.05).

### ANCOVA assessment of left ventricular function among different obesity phenotypes (Table 3)

**Table 3:** Left Ventricular Functional Parameters in Different Obesity Phenotypes

	MHNW	MHO	MUNW	MUO	*p*-value
LVEF, %	67.79 ± 4.33	67.74 ± 4.16	67.43 ± 3.77	67.58 ± 4.73	0.979
LVGLS, %	-19.49 ± 1.91	-19.10 ± 1.68	-16.77 ± 1.77^*a^	-15.53 ± 1.73^*ab^	< 0.001
E/A	0.95 ± 0.03	0.91 ± 0.04	0.81 ± 0.04^*a^	0.73 ± 0.03^*^	< 0.01
e’, m/s	7.47 ± 0.21	6.96 ± 0.23	6.54 ± 0.29^*^	5.64 ± 0.22^*a^	< 0.01
LASr, %	35.41 ± 0.92	33.79 ± 1.16	30.91 ± 1.24^*^	30.56 ± 0.98^*^	< 0.01

Adjusted factors: age, sex, smoking history, alcohol consumption history, and estimated glomerular filtration rate. *Abbreviations*: *LVEF* Left Ventricular Ejection Fraction, *LVGLS* Left Ventricular Global Longitudinal Strain, *E* Early Diastolic Mitral Inflow Velocity, *A* Late Diastolic Mitral Inflow Velocity, *e*’ Average Early Diastolic Mitral Annular Velocity (septal and lateral), *LASr*: Left Atrial Reservoir Strain. Significance markers: *: Significant difference vs. MHNW (*P* < 0.05); a: Significant difference vs. MHO (*P* < 0.05); b: Significant difference vs. MUNW (*P* < 0.05)

ANCOVA was performed for LVEF and LVGLS across different obesity phenotypes, adjusting for age, sex, smoking status, alcohol consumption, and eGFR as covariates. The results demonstrated that LVGLS remained significantly lower in the MUNW and MUO compared with the control group (*P* < 0.01), while LVEF showed no significant differences among the groups (*P* > 0.05).

After adjustment for covariates, significant differences persisted between the control group and MUNW and MUO groups in e’, E/A, LASr, and LAScd (*P* < 0.01). In contrast, differences between the control group and MHO group were no longer statistically significant.

### MW ANCOVA analysis (Table 4; Fig. 2, Additional file 1)

**Table 4:** Global Myocardial Work Parameters in Different Obesity Phenotypes

	MHNW	MHO	MUNW	MUO	*p*-value
GCW, mmHg	2413.1 ± 45.6	2334.7 ± 57.5	2074.5 ± 61.5^*a^	2128.3 ± 48.4^*a^	< 0.01
GWW, mmHg	90.2 ± 7.6	98.9 ± 9.5	119.4 ± 10.2^*^	170.6 ± 8.0^*ab^	< 0.01
GWE, %	95.6 ± 0.3	95.2 ± 0.4	93.4 ± 0.4^*a^	91.3 ± 0.3^*ab^	< 0.01
GWI, mmHg	2150.1 ± 44.3	2026.4 ± 55.8	1777.2 ± 59.7^*a^	1783.0 ± 47.1^*a^	< 0.01

Adjusted factors: age, sex, smoking history, alcohol consumption history, and estimated glomerular filtration rate. *GCW* Global Constructive Work, *GWW* Global Waste Work, *GWE* Global Work Efficiency, *GWI* Global Work Index. Significance markers: *: Significant difference vs. MHNW (*P* < 0.05); a: Significant difference vs. MHO (*P* < 0.05); b: Significant difference vs. MUNW (*P* < 0.05)

**Fig. 2:**
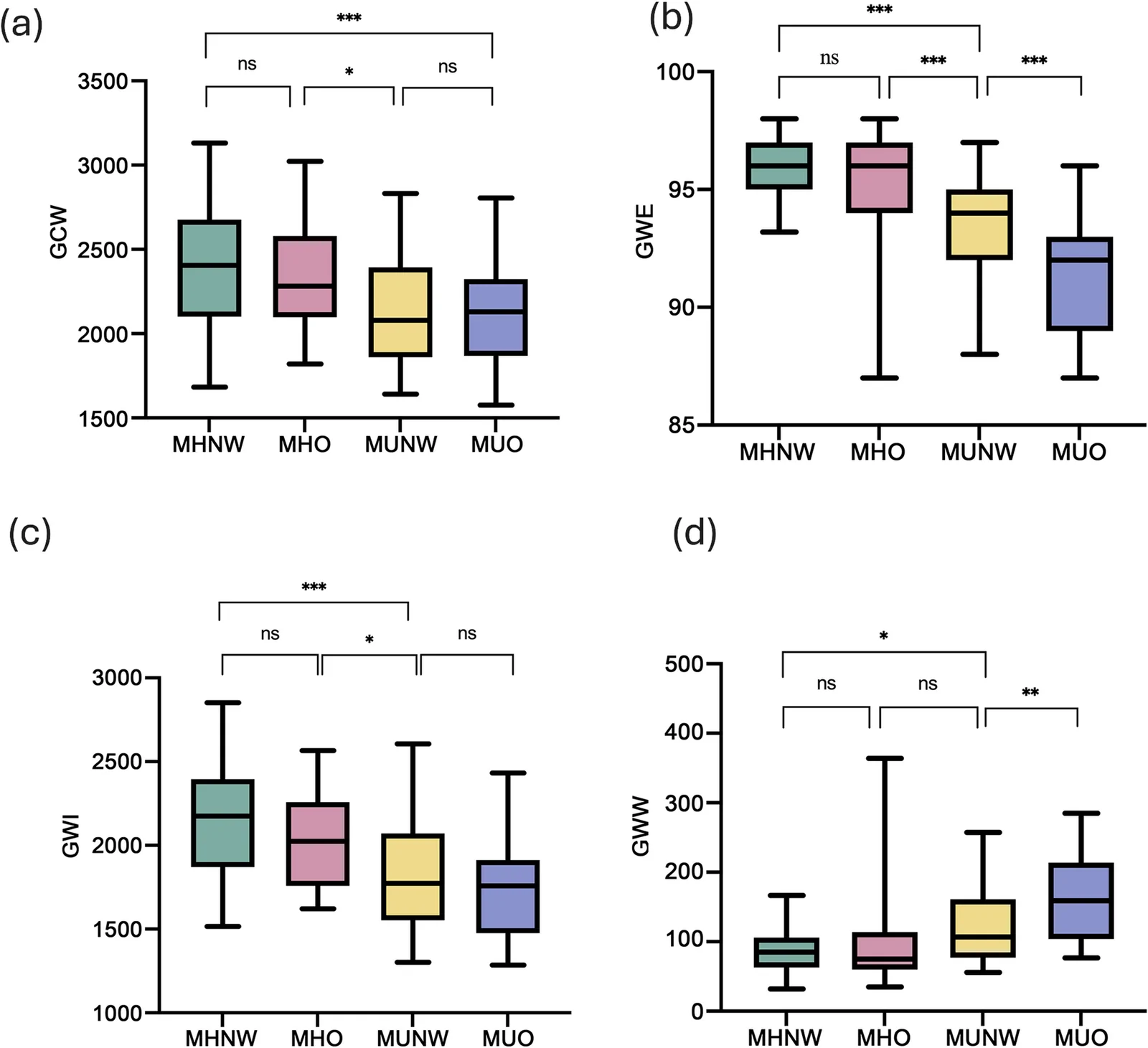
Comparison of Myocardial Work (MW) Parameters Across Different Obesity Phenotypes. (**a**) Comparison of Global Work Efficiency (GWE) across different obesity phenotypes (**b**) Comparison of Global Constructive Work (GCW) across different obesity phenotypes (**c**) Comparison of Global Waste Work (GWW) across different obesity phenotypes (**d**) Comparison of Global Work Index (GWI) across different obesity phenotypesAbbreviations: GCW: Global Constructive Work; GWW: Global Waste Work; GWE: Global Work Efficiency; GWI: Global Work Index. *: *P* < 0.05, **: *P* < 0.01, ***: *P* < 0.001

After adjusting for age, sex, smoking status, alcohol consumption, and eGFR, significant differences were observed between the control group and obesity phenotype groups in all global myocardial work parameters (GWI, GCW, GWW, and GWE) (*P* < 0.01). The MHNW exhibited the highest GWE, GWI, and GCW, and the lowest GWW. Significant differences in GWE were observed among different obesity phenotypes (*P* < 0.05). Within the MH population, no significant differences in any MW parameters were observed between the MHO and MHNW. Compared with the MU population, MH individuals had higher GWE, GWI, and GCW, and lower GWW.

Pairwise comparisons among the three obesity phenotypes revealed that the MUO had the lowest GWE and highest GWW. GWI and GCW were lowest in the MUNW and MUO. The MHO had the highest GWI, GCW, and GWE. GWW did not differ significantly between the MHO and MUNW.

To confirm that the observed MW impairments were not solely driven by hemodynamic afterload differences across groups, a sensitivity analysis was performed. Even after rigorously adjusting for continuous SBP and DBP, the significant impairments in MW parameters in the MUO group remained highly robust (Additional file 1).

### ANCOVA assessment of EAT volume among different obesity phenotypes (Table 5; Fig. 3)

**Table 5:** Epicardial Adipose Tissue Volume in Different Obesity Phenotypes

	MHNW	MHO	MUNW	MUO	*p*-value
EAT volume, cm^3^	50.44 ± 1.63	65.24 ± 2.05^*^	63.65 ± 2.19^*^	70.59 ± 1.73^*b^	< 0.01
EAT volume index, cm^3^/m^2^	32.56 ± 0.90	37.49 ± 1.13^*^	38.71 ± 1.21^*^	39.32 ± 0.96^*^	< 0.01

Adjusted factors: age, sex, smoking history, alcohol consumption history, and estimated glomerular filtration rate. EAT volume: Epicardial adipose tissue volume; EAT volume index: Epicardial adipose tissue volume adjusted for body surface area. Significance markers: *: Significant difference vs. MHNW (*P* < 0.05), b: Significant difference vs. MUNW (*P* < 0.05)

**Fig. 3:**
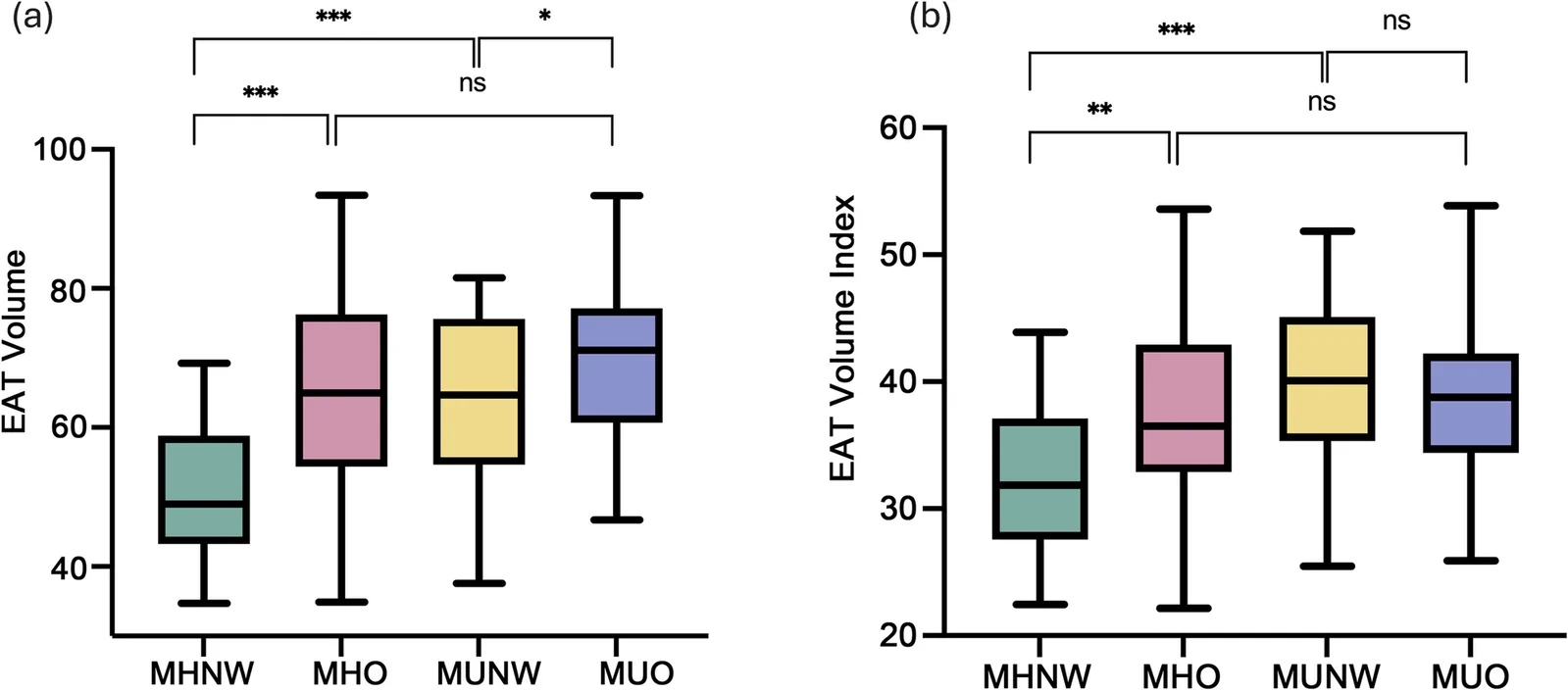
Comparison of Epicardial Adipose Tissue (EAT) Volume Across Different Obesity Phenotypes. Abbreviations: EAT volume index: Epicardial adipose tissue volume adjusted for body surface area. *: *P* < 0.05, **: *P* < 0.01, ***: *P* < 0.001

Compared with the control group, EAT volume was significantly increased in the MHO, MUNW, and MUO (*P* < 0.01). The MUO exhibited a significantly larger EAT volume than the MUNW (*P* < 0.01). No significant differences in EAT volume were observed between the MHO and either the MUNW or MUO.

After adjustment for BSA, the differences in EAT volume index between the MHNW and the other three groups remained significant (*P* < 0.01). However, the differences between the MUO and MUNW were no longer statistically significant.

### Univariate and multivariate linear regression analysis of MW parameters in different obesity phenotypes (Tables 6, 7, 8, 9, and 10)

**Table 6:** Multiple Linear Regression Analysis of Myocardial Work Parameters

	β	SE	95%CI	*P*
GWE				
Crude	-3.467	0.349	(-4.151, -2.783)	< 0.001
Model 1	-3.346	0.355	(-4.042, -2.649)	< 0.001
Model 2	-3.023	0.374	(-3.756, -2.289)	< 0.001
Model 3	-2.979	0.392	(-3.748, -2.210)	< 0.001
Model 4	-2.964	0.400	(-3.747, -2.181)	< 0.001
GWW				
Crude	59.301	9.062	(77.062,41.541)	< 0.001
Model 1	56.266	8.977	(73.861,38.671)	< 0.001
Model 2	44.734	9.181	(62.732,26.742)	< 0.001
Model 3	46.706	9.803	(65.920,27.493)	< 0.001
Model 4	47.698	9.973	(67.246,28.151)	< 0.001
GCW				
Crude	-264.415	54.008	(-370.270, -158.560)	< 0.001
Model 1	-249.658	51.487	(-350.571, -148.745)	< 0.001
Model 2	-252.046	54.687	(-359.230, -144.861)	< 0.001
Model 3	-254.392	57.100	(-366.305, -142.478)	< 0.001
Model 4	-243.064	57.940	(-356.625, -129.504)	< 0.001
GWI				
Crude	-314.916	51.738	(-416.322, -213.511)	< 0.001
Model 1	-291.861	49.902	(-389.667, -194.055)	< 0.001
Model 2	-275.737	52.884	(-379.387, -172.087)	< 0.001
Model 3	-264.201	56.123	(-374.201, -154.201)	< 0.001
Model 4	-259.125	57.108	(-371.056, -147.194)	< 0.001

*Crude* Univariate regression with MUH as the independent variable, Model 1: Crude + age + sex + smoking history + alcohol consumption history; Model 2: Model 1 + BMI; Model 3: Model 2 + estimated glomerular filtration rate + glycated hemoglobin + N-terminal pro–B-type natriuretic peptide + left ventricular mass; Model 4: Model 3 + epicardial adipose tissue volume. *GWE* Global Work Efficiency, *GWW* Global Waste Work, *GCW* Global Constructive Work, *GWI* Global Work Index, *BMI* Body Mass Index, β Coefficient for MUH, *P* Significance of MUH

**Table 7:** GWE and Different Clinical Parameters

	β	SE	95%CI	*P*
MUH	-2.964	0.400	(-2.181, -3.747)	< 0.001
Female	-0.21	0.501	(-1.192,0.772)	0.772
Age	-0.043	0.020	(-0.083, -0.003)	0.034
BMI	-0.184	0.064	(-0.309, -0.059)	0.004
eGFR	0.002	0.016	(-0.029,0.033)	0.892
HbA1c	0.252	3.379	(-0.017,0.521)	0.066
NT-proBNP	-0.001	0.004	(-0.010,0.007)	0.777
LVM	-0.008	0.006	(-0.020,0.004)	0.210
Never smoking	0.538	0.286	(-0.449,1.525)	0.286
Drinking	-0.378	0.354	(-1.625,0.869)	0.552
EAT volume	-0.003	0.829	(-0.031,0.025)	0.829

*Abbreviations*: *GWE* Global Work Efficiency, *MUH* Metabolically Unhealthy, *BMI* Body Mass Index, *eGFR* Estimated Glomerular Filtration Rate, *HbA1c* Glycated Hemoglobin, *NT-proBNP* N-terminal pro–B-type Natriuretic Peptide, *LVM* Left Ventricular Mass, *EAT* Epicardial Adipose Tissue

**Table 8:** GWW and Different Clinical Parameters

	β	SE	95%CI	*P*
MUH	47.698	9.973	(67.246,28.151)	< 0.001
Female	26.839	12.51	(2.32,51.358)	0.032
Age	1.941	0.506	(0.949,2.933)	< 0.001
BMI	4.247	1.592	(1.126,7.368)	0.008
eGFR	0.087	0.394	(-0.685,0.860)	0.697
HbA1c	-8.428	3.426	(-15.143, -1.713)	0.014
NT-proBNP	-0.017	0.109	(-0.241,0.189)	0.813
LVM	0.357	0.152	(0.060,0.065)	0.019
Never smoking	-28.980	12.575	(-53.626, -4.334)	0.021
Drinking	12.945	15.880	(-18.180,44.069)	0.415
EAT volume	-0.206	0.361	(-0.914,0.501)	0.567

*Abbreviations*: *GWW* Global Waste Work, *MUH* Metabolically Unhealthy, *BMI* Body Mass Index, *eGFR* Estimated Glomerular Filtration Rate, *HbA1c* Glycated Hemoglobin, *NT-proBNP* N-terminal pro–B-type Natriuretic Peptide, *LVM* Left Ventricular Mass, *EAT* Epicardial Adipose Tissue

**Table 9:** GCW and Different Clinical Parameters

	β	SE	95%CI	*P*
MUH	-243.064	57.940	(-356.625, -129.504)	< 0.001
Female	230.708	72.675	(88.267,373.148)	0.002
Age	-12.306	2.941	(-18.07, -6.541)	< 0.001
BMI	-6.221	9.251	(-24.354,11.911)	0.501
eGFR	2.510	2.781	(-2.941,7.961)	0.367
HbA1c	-55.757	19.903	(-94.767, -16.748)	0.005
NT-proBNP	-0.883	0.944	(-2.732,0.967)	0.350
LVM	3.128	0.881	(1.401,4.854)	< 0.001
Never smoking	-141.429	91.152	(-320.083,37.225)	0.121
Drinking	4.974	111.792	(-214.134,224.082)	0.965
EAT volume	-4.125	2.765	(-9.545,1.295)	0.136

*Abbreviations*: *GCW* Global Constructive Work, *MUH* Metabolically Unhealthy, *BMI* Body Mass Index, *eGFR* Estimated Glomerular Filtration Rate, *HbA1c* Glycated Hemoglobin, *NT-proBNP* N-terminal pro–B-type Natriuretic Peptide, *LVM* Left Ventricular Mass, *EAT* Epicardial Adipose Tissue

**Table 10:** GWI and Different Clinical Parameters

	β	SE	95%CI	*P*
MUH	-259.125	57.108	(-371.056, -147.194)	< 0.001
Female	204.037	71.632	(72.474,419.415)	0.004
Age	-8.638	2.899	(-14.320, -2.956)	0.003
BMI	-12.784	9.119	(-30.656,5.089)	0.161
eGFR	1.442	2.609	(-3.671,6.555)	0.580
HbA1c	-53.926	19.617	(-92.375, -15.477)	0.006
NT-proBNP	-0.284	0.885	(-2.019,1.451)	0.748
LVM	2.143	0.868	(0.442,3.845)	0.016
Never smoking	-39.706	85.505	(-207.292,127.881)	0.642
Drinking	64.603	104.866	(-140.931,270.138)	0.538
EAT volume	-2.450	2.594	(-7.534,2.635)	0.345

*Abbreviations*: *GWI* Global Work Index, *MUH* Metabolically Unhealthy, *BMI* Body Mass Index, *eGFR* Estimated Glomerular Filtration Rate, *HbA1c* Glycated Hemoglobin, *NT-proBNP* N-terminal pro–B-type Natriuretic Peptide, *LVM* Left Ventricular Mass, *EAT* Epicardial Adipose Tissue

Univariate linear regression analysis showed that GWE, GWW, GCW, and GWI were all significantly associated with MUH (*P* < 0.001). Based on these results, additional clinically relevant variables that differed between groups and were closely associated with left ventricular function were sequentially included as covariates to construct multivariate linear regression models. Model 1 included age, sex, smoking status, and alcohol consumption in addition to MUH. Model 2 further added BMI. Model 3 additionally incorporated eGFR, NT-proBNP, HbA1c, and LVM. Model 4 included EAT volume based on Model 3.

Because the criteria for MUH already encompass multiple parameters such as blood pressure, FPG, HDL-C, TG, and WC, these variables were not included as covariates in the regression models. Even after sequentially adjusting for potential confounders, MUH remained significantly associated with MW parameters in Model 4: GWE (β = −2.964), GWW (β = 47.698), GCW (β = −243.064), and GWI (β = −259.125) (*P* < 0.001). Additionally, age and BMI were independent determinants of impaired GWE (β = −0.043, *P* < 0.05; β = −0.184, *P* < 0.01) and GWW (β = 1.941, *P* < 0.001; β = 4.247, *P* < 0.01). Female, HbA1c, and LVM were independently associated with GWW (β = 26.839, − 8.428, 0.357), GCW (β = 230.708, − 55.757, 3.128), and GWI (β = 204.037, − 53.926, 2.143) (*P* < 0.05). EAT volume was not independently associated with any MW parameter (*P* > 0.05).

### Moderating effect of eat volume on the relationship between metabolic unhealthiness and myocardial work (Tables 11 and 12; Fig. 4)

**Table 11:** Modulatory Effect of EAT Volume on MUH and Myocardial Work Parameters

	GWE	GWW	GCW	GWI
Main Effect				
MUH	-2.81 (0.37) ^**^	45.4 (9.42) ^**^	-243 (57.8) ^**^	-259 (57.0) ^**^
EAT volume	-0.01 (0.01)	-0.12 (0.35)	-2.28 (2.07)	-1.17 (2.04)
Modulatory Effect				
MUH × EAT volume	-0.06 (0.02) ^**^	1.37 (0.63) ^*^	3.19 (3.69)	-2.69 (3.64)
P for Interaction	< 0.01 ^**^	0.02 ^*^	0.39	0.46

Covariates including age, sex, body mass index, smoking history, alcohol consumption history, glycated hemoglobin, N-terminal pro–B-type natriuretic peptide, estimated glomerular filtration rate, and left ventricular mass were included in the regression model. *EAT* Epicardial Adipose Tissue, *MUH* Metabolically Unhealthy, *MW* Global Myocardial Work, *P* for Interaction, Significance of the modulatory effect; Significance markers, *: *P* < 0.05, **: *P* < 0.01

**Table 12:** Slope Analysis of MUH on Myocardial Work at Different Levels of EAT Volume

	EAT Volume	β	SE	t value	*P*
GWE	Low Level	-1.92	0.55	-3.49	< 0.001
	Median	-2.94	0.39	-7.5	< 0.001
	High Level	-3.97	0.54	-7.38	< 0.001
GWW	Low Level	25.37	13.77	1.84	0.07
	Median	47.27	9.86	4.8	< 0.001
	High Level	69.17	13.51	5.12	< 0.001

*Abbreviations*: *GWE* Global Work Efficiency, *GWW* Global Waste Work

**Fig. 4:**
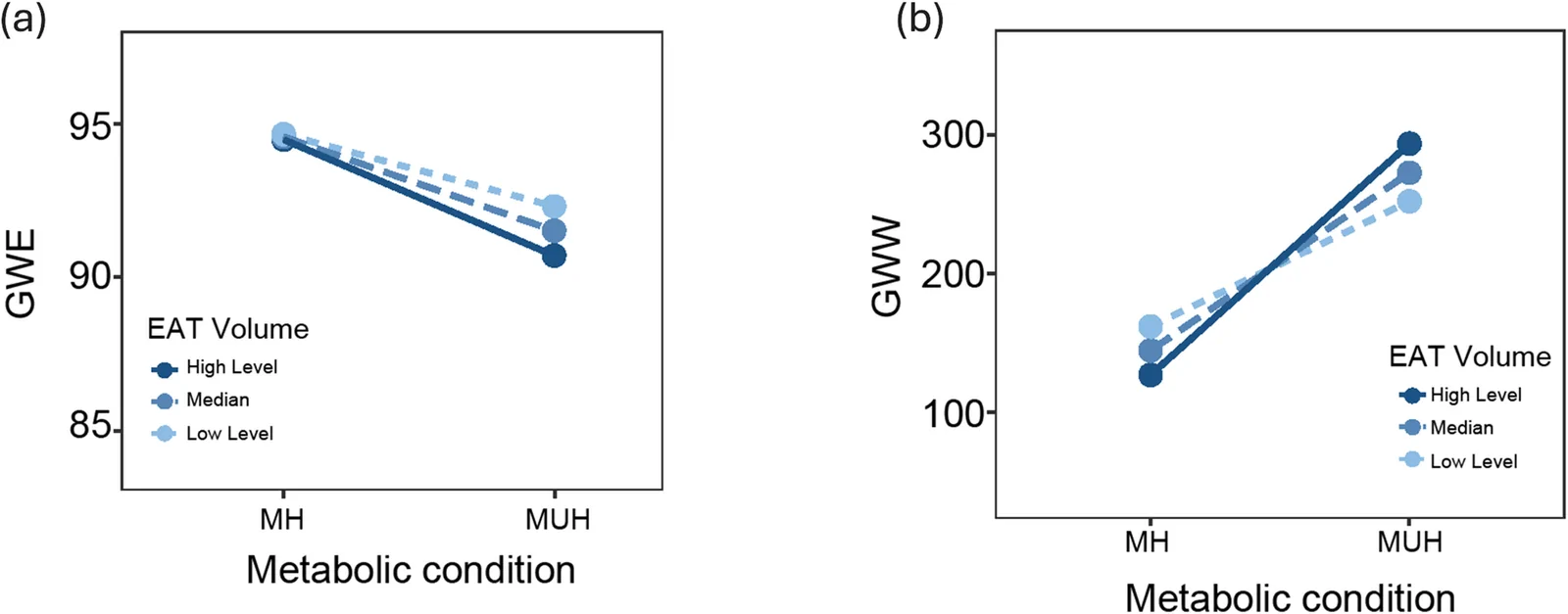
Moderation effects of GWE and GWW low level: 46.6 cm³, median: 61.6 cm³, high level: 76.6 cm

Building on Model 4, the interaction term between MUH and EAT volume was included in the regression equations to investigate whether EAT volume moderates the relationship between MUH and GWE or MUH and GWW. The results showed that the main effects of MUH on GWE and GWW were significant (β = −2.814 and 45.370, *P* < 0.001). Although the main effects of EAT on GWE and GWW were not significant (*P* > 0.05), the interaction terms (MUH × EAT volume) were significant for both GWE and GWW (β = −0.062 and 1.372, *P* < 0.05), with F values of 5.79 and 4.08 (*P* < 0.05). No significant moderating effects of EAT volume were observed on the relationship between MUH and LVGLS, GCW or GWI (*P* > 0.05).

To make this interaction clinically interpretable, simple slope analyses were performed at three levels of EAT volume. For GWE, the detrimental effect of MUH was most pronounced at high EAT volume (β = -3.97, *P* < 0.001), remaining significant at medium EAT (β = -2.94, *P* < 0.001) and low EAT volumes (β = -1.92, *P* < 0.001). Correspondingly, the association between MUH and elevated GWW was markedly amplified at high EAT volume (β = 69.17, *P* < 0.001) compared to medium EAT volume (β = 47.27, *P* < 0.001). Notably, at low EAT volume, the impact of MUH on GWW was blunted and non-significant (β = 25.37, *P* = 0.070). These results quantitatively demonstrate that EAT expansion is a critical exacerbating factor for the subclinical mechanical impairment induced by metabolic unhealthiness.

### Sensitivity analysis with WHR adjustment (additional file 2; additional file 3; additional file 4)

To account for the potential confounding effect of visceral adiposity, a sensitivity analysis adjusting for WHR instead of BMI was performed. As presented in Additional file 2 − 1 to 2–5, the detrimental main effects of MUH status on all four myocardial work parameters remained highly robust (*P* < 0.001 for all models), whereas WHR itself was not significantly associated with these parameters (all *P* > 0.05). Furthermore, the modulatory effect of EAT volume on GWE and GWW was well preserved even after adjusting for WHR (Additional file 3 and Additional file 4), confirming that the local pathogenic impact of EAT expansion is independent of generalized visceral adiposity.

### Inter-observer and intra-observer variability

GWE, GWW, GCW, and GWI showed excellent intra- and inter-observer reliability (all ICCs > 0.85).

## Discussion

The present study demonstrated that the use of MW technology detected subclinical cardiac dysfunction in individuals with MUNW and MUO, whereas the MHO did not exhibit early cardiac functional impairment despite the presence of metabolic risk factor alterations. Group comparisons indicated that subclinical left ventricular dysfunction was more pronounced in the MUO than in the MUNW.

Obesity is an independent risk factor for cardiovascular disease. However, BMI-based definitions of obesity overlook the distinct physiological and pathophysiological roles of regional fat distribution. This limitation may explain why certain individuals with higher BMI but specific metabolic characteristics paradoxically exhibit lower cardiovascular risk in prospective cohort studies. Obesity phenotype stratification facilitates precise risk assessment and improves cardiovascular risk prediction.

### Assessment of subclinical cardiac dysfunction: LVEF, LVGLS, and myocardial work

While LVEF undeniably remains the cornerstone and most practical parameter for evaluating cardiac function in routine clinical practice, its sensitivity is often insufficient for detecting early, subclinical myocardial impairment. In the present study, both LVGLS and MW parameters detected varying degrees of impairment despite preserved LVEF, demonstrating greater sensitivity in identifying early cardiac dysfunction. Because a substantial proportion of patients with HBP and MetS were included in this study, MW offers advantages over LVGLS by better accounting for afterload effects. Furthermore, MW offers insights into myocardial oxygen consumption and ventricular coordination, with reliable measurements across observers. Therefore, it is better for evaluating subclinical cardiac dysfunction in diverse metabolic groups.

Huang et al. [12] reported that elevated systolic blood pressure in patients with higher BMI led to increases in GWI and GCW. Early blood pressure elevation may result in proportional increases in GCW and GWW without affecting GWE [20]. Accordingly, GWE may represent the most appropriate MW parameter for evaluating subclinical cardiac dysfunction across different obesity phenotypes.

### Metabolic features and cardiovascular risk of MHO and MUNW individuals

Although traditionally considered benign, MHO carries a higher long-term cardiovascular risk than MHNW. It frequently acts as a transitional phase, wherein **e**arly subclinical changes—such as elevated branched-chain amino acids and apolipoprotein B [21, 22]—predispose approximately one-third of these individuals to eventually progress into a metabolically unhealthy state [24]. Similarly, in our study, MHO individuals exhibited lower HDL-C levels than MHNW, which is associated with increased future cardiovascular event risk [22]. Additionally, MHO participants had significantly higher WC and WHR than MHNW, consistent with characteristics of metabolically unhealthy populations. Therefore, although MW parameters in MHO did not differ significantly from the control group in early stages, alterations in metabolic risk factors indicate that this population is more prone to progression toward MUO. Early lifestyle interventions targeting MHO individuals are particularly important to prevent progression to higher cardiovascular risk.

Among normal-weight individuals, lower percentages of gluteofemoral and leg fat are the strongest predictors of metabolically unhealthy status [23] and can independently predict cardiometabolic disease risk [24]. Given the same total body fat, relatively lower gluteofemoral and leg fat is a characteristic fat distribution pattern in MUNW individuals. Impaired proliferation and expansion of subcutaneous adipose tissue lead to chronic dysfunction of fat storage in white adipose tissue, resulting in ectopic fat deposition [25]. Danforth et al. [26] confirmed that failure of subcutaneous fat to expand normally contributes to increased cardiometabolic risk. In our study, compared with MHNW, the MUNW group had significantly higher WC and WHR despite similar BMI; compared with MHO, WC was lower but WHR was higher, and compared with MUO, WHR showed no significant difference. After adjusting for BSA, the EAT volume index was comparable between MUNW and MUO, indicating substantial ectopic fat accumulation in MUNW and elevated cardiovascular risk.

### Subclinical cardiac dysfunction in MUNW individuals

The MUNW group exhibited pronounced subclinical cardiac dysfunction. In terms of MW parameters, this was reflected by reduced GCW, GWI, and GWE, along with increased GWW. On one hand, Huang et al. [27] reported that patients with type 2 diabetes mellitus had lower GLS, GCW, and GWI compared with controls, while GWW and GWE showed no significant differences. In our study, compared with metabolically healthy groups, MUNW individuals had higher fasting glucose and marked insulin resistance. Additionally, the measured IVSd in MUNW patients was significantly higher than that in the MHNW controls, indicating the presence of early myocardial remodeling. The structural adaptation and potential increase in GCW induced by afterload elevation were insufficient to compensate for the decrease in myocardial contractility and GCW caused by impaired mitochondrial excitation–contraction coupling associated with insulin resistance, resulting in overall reductions in GWI and GCW.

Furthermore, decreased GWE reflects mechanical dyssynchrony [28]. In metabolically unhealthy states, adipose inflammation disrupts adiponectin-leptin ratios [29]. This imbalance, alongside hyperglycemia, promotes left ventricular hypertrophy and myocardial fibrosis, ultimately causing heterogeneous conduction and mechanical dyssynchrony. Consequently, MUNW individuals exhibited left ventricular mechanical dyssynchrony, reflected by increased GWW and decreased GWE. Given that they exhibit significant early subclinical cardiac dysfunction, early identification of metabolically unhealthy individuals within normal-BMI populations is critical for timely intervention.

### Cardiac dysfunction and the role of EAT in MUO individuals

The MUO group was relatively young, likely because older MUO individuals frequently progress to overt cardiac dysfunction and were thus excluded from the study. Despite being younger, they exhibited higher adiposity indices, dyslipidemia, and greater left ventricular mass, indicating that insulin resistance and ectopic fat are closely associated with pronounced left ventricular impairment.

Regarding MW parameters, MUO individuals displayed marked mechanical dyssynchrony with the highest GWW and lowest GWE. Notably, GWI was not further reduced in MUO compared to MUNW. This is likely because elevated blood pressure and pronounced myocardial hypertrophy in MUO increase afterload, which upwardly shifts the pressure-strain loop and overestimates GWI. These findings suggest that GWI may not be the optimal index for detecting subclinical cardiac dysfunction across different obesity phenotypes.

Epicardial adipose tissue (EAT) volume is associated with impaired adipocyte differentiation, myocardial fibrosis, elevated left ventricular filling pressures, and systemic inflammation. Prior prospective studies incorporating EAT into multivariable models identified it as an independent predictor of LVGLS, independent of sex, age, or insulin resistance [16]. In the present study, all three obesity phenotypes exhibited significantly increased EAT volume. Importantly, our multivariable analysis demonstrated that EAT volume was not an independent determinant of MW parameters in the overall cohort. Instead, the relationship between EAT and subclinical cardiac dysfunction appears to be highly context-dependent. The significant interaction effect indicates that increased EAT volume is associated with more pronounced impairment of MW parameters, and this association exists only in the presence of systemic metabolic dysfunction (MUH).

In metabolic syndrome, insulin resistance and oxidative stress impair myocardial contractility, leading to decreased GWI and GCW. Other studies have shown that obesity, hyperglycemia, and hypertension can induce left ventricular dyssynchrony, resulting in increased GWW in metabolically unhealthy individuals. Multivariate regression confirmed that after adjusting for age, sex, and BMI, MUH status was independently associated with lower GWE, GCW, and GWI, as well as higher GWW.

Interaction analysis between EAT volume and MUH revealed that increased EAT was associated with more pronounced reductions in GWE and elevations in GWW in MUH individuals, independent of BMI. After ruling out multicollinearity (VIF < 5), we found a robust, positive moderating effect of EAT volume on MUH. In metabolically unhealthy individuals, EAT expansion synergistically worsened mechanical dyssynchrony (decreased GWE and increased GWW), but did not significantly alter GCW or GWI. Previous studies have shown that HFrEF patients have lower EAT volume than controls or HFpEF patients [30, 31]. EAT expansion may not directly impair myocardial contractility or affect useful work (GCW). GWE, which reflects the proportion of useful work relative to total work, may be influenced by EAT primarily through its effect on wasted work (GWW). Furthermore, our study quantified EAT solely by CT volume, which captures only one biological dimension of cardiac adiposity. A comprehensive characterization of EAT benefits from integrating complementary modalities, such as CT attenuation, echocardiography, and cardiac magnetic resonance [32]. Future studies should incorporate multimodal approaches including CMR T1 mapping or late gadolinium enhancement (LGE) to comprehensively evaluate EAT and explore the biological link between EAT accumulation and cardiac mechanical dyssynchrony.

In our study, elevated GWW correlated with age, BMI, HbA1c, and LVM. Increased myocardial EAT infiltration may augment LVM and physically impede conduction. Furthermore, EAT-secreted pro-inflammatory factors during metabolic impairment exacerbate oxidative stress and abnormal coupling, which may be associated with amplified mechanical dyssynchrony and GWW independently of BMI. Impaired subcutaneous fat storage in metabolically unhealthy individuals leads to accumulation of visceral fat, increasing WC and WHR, with excess fat eventually depositing in the heart, liver, and skeletal muscle. Importantly, our sensitivity analysis demonstrated that the independent impact of MUH and its adverse interaction with EAT volume on myocardial work persist even after rigorously adjusting for WHR. This reinforces the concept that EAT exerts a localized, paracrine toxic effect on the myocardium that cannot be explained solely by systemic visceral fat accumulation.

### Limitations

Thus, in MUH individuals, even in the absence of overt cardiac dysfunction, EAT volume should be considered a useful metric for assessing potential cardiac risk. This study has several limitations. First, this was a single-center study with a relatively small sample size. Although collinearity diagnostics confirmed the stability of our multivariable models, the modest subgroup sizes may limit the overall statistical power and precision, particularly for detecting complex interaction effects. Given that metabolic syndrome comprises varying combinations of hypertension, dyslipidemia, and impaired glucose tolerance, future studies with larger cohorts and more refined subgroup analyses are warranted to distinguish between overweight and obese populations and to dissect the specific impacts of distinct metabolic risk factor clusters on myocardial work parameters. Meanwhile, due to the multiple statistical tests performed, the possibility of type I error exists. Therefore, findings from subgroup and interaction analyses should be interpreted cautiously and require further validation.

Second, the cross-sectional design precludes causal inference and lacks longitudinal outcome data. Therefore, the prognostic significance of the observed subclinical MW impairment requires further prospective validation. Additionally, this single-time-point assessment limits our ability to track the known progression from MHO to a metabolically unhealthy state. Third, despite adjustment for age, residual confounding related to biological aging may still exist. In particular, sarcopenic obesity, a condition commonly observed in older individuals and characterized by reduced muscle mass and muscle strength, was not specifically assessed in this study. Therefore, its potential impact on the observed associations cannot be excluded. Similarly, age can also affect sex hormone levels in older women, thereby further altering ectopic fat accumulation (including EAT), which represents another limitation imposed by age. Fourth, selection bias may exist because EAT volume was assessed using coronary CTA. Therefore, only patients with clinical indications for CT examination were included, which may limit the generalizability of our findings to the general population.

### Clinical Implications

The present study provides several clinically relevant insights. First, significant differences in myocardial work (MW) parameters were observed despite preserved LVEF, suggesting that MW may provide additional information for identifying subclinical myocardial dysfunction across different obesity phenotypes. Therefore, MW may complement conventional echocardiographic assessment when evaluating early myocardial impairment.

Second, our findings indicate that metabolic health status appears to be more closely associated with myocardial mechanical dysfunction than BMI alone. In particular, individuals with the metabolically unhealthy normal-weight (MUNW) phenotype exhibited substantial impairment in myocardial work parameters despite having a normal BMI. These findings highlight the importance of incorporating metabolic phenotyping into cardiovascular risk assessment rather than relying solely on body size.

Third, although EAT volume was not independently associated with myocardial work parameters after multivariable adjustment, the significant interaction between EAT burden and metabolic unhealthiness suggests that EAT may contribute to identifying individuals who are more vulnerable to myocardial mechanical inefficiency. Assessment of EAT may therefore provide additional value for risk stratification among metabolically unhealthy individuals.

## Conclusion

MW provides a more sensitive and reliable assessment of subclinical cardiac dysfunction than LVEF, allowing for the early detection of impaired cardiac function in individuals with different obesity phenotypes. Our findings indicate a progressive increase in subclinical cardiac impairment from MHO to MUNW and MUO. Furthermore, although EAT volume is not an independent determinant of MW impairment in the overall cohort, increased EAT volume in metabolically unhealthy individuals was associated with a greater reduction in GWE and an elevation in GWW.

## Supplementary Information

Additional file 1.

Additional file 2.

Additional file 3.

Additional file 4.

Additional file 5.

## Data Availability

Deidentified data supporting this study are available from the Corresponding author ( Shaoxin Zheng ) upon reasonable request.

## Abbreviations

BMI: Body Mass Index
BSA: Body Surface Area
DBP: Diastolic blood pressure
EAT: Epicardial adipose tissue
eGFR: Estimated glomerular filtration rate
FPG: Fasting plasma glucose
GCW: Global constructive work
GLP1- RA: Glucagon-like peptide-1 Receptor agonists
GWE: Global work efficiency
GWI: Global work index
GWW: Global waste work
HbA1c: Glycosylated Hemoglobin
HDL-C: High Density Lipoprotein Cholesterol
HF: Heart Failure
HFmrEF: Heart failure with moderately reduced ejection fraction
HFpEF: Heart failure with preserved ejection fraction
LVGLS: Left ventricular global longitudinal strain
IVSd: Interventricular septum thickness at diastole
LAd: Left atrial diameter
LDL-C: Low Density Lipoprotein Cholesterol
LVEDd: Left ventricular end diastolic diameter
LVEF: Left ventricular ejection fraction
LVM: Left ventricular mass
LVMI: Left ventricular mass index
LVPWd: Left ventricular posterior wall during diastole
MetS: Metabolic syndrome
MHNW: Metabolically healthy normal weight
MHO: Metabolically healthy obesity
MUNW: Metabolically unhealthy normal weight
MUO: Metabolically unhealthy obesity
MW: Global Myocardial Work
NT-proBNP: N-terminal brain natriuretic peptide
SBP: Systolic Blood Pressure
SGLT2-i: Sodium-dependent glucose transporters 2 inhibitor
TC: Total Cholesterol
TG: Triglyceride
WC: Waist Circumference
WHR: Waist-Hip Ratio
WHO: World health organization
