# Supplementary material for: Analysis of myocardial work in different obesity phenotypes and its association with epicardial adipose tissue
Source: BMC Cardiovasc Disord. 2026 Jul 22;26:936. doi: 10.1186/s12872-026-06298-3 (PMC13679159; doi:10.1186/s12872-026-06298-3)
Supplement: Supplementary file 1 [file 12872_2026_6298_MOESM1_ESM.pdf]

Additional file 1. Sensitivity Analysis of Global Myocardial Work Parameters

Adjusted for Continuous Blood Pressure

	MHNW	MHO	MUNW	MUO	p-value
GCW, mmHg%	2402.1±47.8	2355.1±54.3	2070.2±54.3[*a]	2008.4±49.4[*a]	< 0.001
GWW, mmHg%	112.2±10.8	122.8±12.3	136.9±12.3	175.2±11.2[*ab]	< 0.001
GWE, %	95.0±0.4	94.6±0.5	92.9±0.5[*a]	91.2±0.5[*ab]	< 0.001
GWI, mmHg%	2134.3±50.2	2042.3±57	1778.5±57.1[*a]	1692.6±51.9[*a]	< 0.001

Adjusted factors: age, sex, smoking history, alcohol consumption history, and estimated glomerular filtration rate,SBP,DBP. GCW: Global Constructive Work; GWW: Global Waste Work; GWE: Global Work Efficiency; GWI: Global Work Index. Significance markers: *: Significant difference vs. MHNW ($P < 0.05$); a: Significant difference vs. MHO ($P < 0.05$); b: Significant difference vs. MUNW ($P < 0.05$).
