# Supplementary material for: Analysis of myocardial work in different obesity phenotypes and its association with epicardial adipose tissue
Source: BMC Cardiovasc Disord. 2026 Jul 22;26:936. doi: 10.1186/s12872-026-06298-3 (PMC13679159; doi:10.1186/s12872-026-06298-3)
Supplement: Supplementary file 2 [file 12872_2026_6298_MOESM2_ESM.pdf]

Additional file 2-1. Multiple Linear Regression Analysis of Myocardial Work Parameters (Adjusted for WHR)

	β	SE	95%CI	P
**GWE**				
Crude	-3.467	0.349	(-4.151, -2.783)	< 0.001
Model S1	-3.346	0.355	(-4.042, -2.649)	< 0.001
Model S2	-3.141	0.407	(-3.944, -2.338)	< 0.001
Model S3	-3.050	0.427	(-3.893, -2.207)	< 0.001
Model S4	-2.979	0.430	(-3.828, -2.130)	< 0.001
**GWW**				
Crude	59.301	9.062	(77.062,41.541)	< 0.001
Model S1	56.266	8.977	(73.861,38.671)	< 0.001
Model S2	51.376	10.286	(71.669, 31.083)	< 0.001
Model S3	47.868	10.579	(68.743, 26.993)	< 0.001
Model S4	47.399	10.703	(68.518, 26.279)	< 0.001
**GCW**				
Crude	-264.415	54.008	(-370.270, -158.560)	< 0.001
Model S1	-249.658	51.487	(-350.571, -148.745)	< 0.001
Model S2	-226.894	59.039	(-343.370, -110.418)	< 0.001
Model S3	-245.074	60.652	(-364.750, -125.398)	< 0.001
Model S4	-234.199	61.082	(-354.728, -113.671)	< 0.001
**GWI**				
Crude	-314.916	51.738	(-416.322, -213.511)	< 0.001
Model S1	-291.861	49.902	(-389.667, -194.055)	< 0.001
Model S2	-248.952	56.982	(-361.370, -136.534)	< 0.001
Model S3	-247.465	59.68	(-365.223, -129.706)	< 0.001
Model S4	-240.142	60.258	(-359.046, -121.238)	< 0.001

Crude: Univariate regression with MUH as the independent variable; Model S1: Crude + age + sex + smoking history + alcohol consumption history; Model S2: Model S1 + WHR; Model S3: Model S2 + estimated glomerular filtration rate + glycated hemoglobin + N-terminal pro–B-type natriuretic peptide + left ventricular mass; Model S4: Model S3 + epicardial adipose tissue volume. GWE: Global Work Efficiency; GWW: Global Waste Work; GCW: Global Constructive Work; GWI: Global Work Index; BMI: Body Mass Index. β: Coefficient for MUH; P: Significance of MUH.

Additional file 2-2. GWE and Different Clinical Parameters (Adjusted for WHR)

	β	SE	95%CI	P
MUH	-2.979	0.43	(-3.828, -2.130)	< 0.001
Female	-0.487	0.52	(-1.514, 0.539)	0.35
Age	-0.031	0.02	(-0.071, 0.008)	0.12
WHR	-1.944	3.37	(-8.593, 4.705)	0.565
eGFR	0.004	0.016	(-0.028, 0.036)	0.813
HbA1c	0.265	0.14	(-0.012, 0.543)	0.06
NT-proBNP	-0.001	0.004	(-0.009, 0.009)	0.968
LVM	-0.013	0.006	(-0.024, -0.001)	0.035
Never smoking	0.553	0.49	(-0.413, 1.519)	0.26
Drinking	-0.299	0.648	(-1.579, 0.980)	0.645
EAT volume	-0.017	0.014	(-0.044, 0.010)	0.22

Abbreviations: GWE: Global Work Efficiency; MUH: Metabolically Unhealthy; WHR: Waist-Hip Ratio; eGFR: Estimated Glomerular Filtration Rate; HbA1c: Glycated Hemoglobin; NT-proBNP: N-terminal pro–B-type Natriuretic Peptide; LVM: Left Ventricular Mass; EAT: Epicardial Adipose Tissue.

Additional file 2-3. GWW and Different Clinical Parameters (Adjusted for WHR)

	β	SE	95%CI	P
MUH	47.399	10.703	(26.279, 68.518)	< 0.001
Female	33.886	12.932	(8.367, 59.405)	0.012
Age	1.941	0.506	(0.949,2.933)	< 0.001
WHR	57.9	83.791	(-107.440, 223.240)	0.491
eGFR	0.053	0.401	(-0.739, 0.844)	0.895
HbA1c	-8.737	3.493	(-15.630, -1.845)	0.013
NT-proBNP	-0.075	0.11	(-0.292, 0.142)	0.494
LVM	0.47	0.147	(0.179, 0.761)	0.002
Never smoking	-28.567	12.174	(-52.588, -4.545)	0.023
Drinking	10.895	16.124	(-20.921, 42.712)	0.5
EAT volume	0.111	0.341	(-0.562, 0.785)	0.745

Abbreviations: GWW: Global Waste Work; MUH: Metabolically Unhealthy; WHR: Waist-Hip Ratio; eGFR: Estimated Glomerular Filtration Rate; HbA1c: Glycated Hemoglobin; NT-proBNP: N-terminal pro–B-type Natriuretic Peptide; LVM: Left Ventricular Mass; EAT: Epicardial Adipose Tissue.

Additional file 2-4. GCW and Different Clinical Parameters (Adjusted for WHR)

	β	SE	95%CI	P
MUH	-234.199	61.082	(-354.728, -113.671)	<0.001
Female	213.51	73.806	(67.874, 359.146)	0.004
Age	12.681	2.85	(7.057, 18.305)	<0.001
WHR	-296.873	478.199	(-1240.470, 646.724)	0.536
eGFR	1.645	2.289	(-2.871, 6.162)	0.473
HbA1c	-53.607	19.935	(-92.944, -14.271)	0.008
NT-proBNP	-0.809	0.628	(-2.047, 0.430)	0.199
LVM	2.982	0.841	(1.323, 4.641)	<0.001
Never smoking	-12.14	69.475	(-149.231, 124.951)	0.862
Drinking	-74.905	92.021	(-256.483, 106.673)	0.417
EAT volume	-2.578	1.948	(-6.421, 1.266)	0.187

Abbreviations: GCW: Global Constructive Work; MUH: Metabolically Unhealthy; WHR: Waist-Hip Ratio; eGFR: Estimated Glomerular Filtration Rate; HbA1c: Glycated Hemoglobin; NT-proBNP: N-terminal pro–B-type Natriuretic Peptide; LVM: Left Ventricular Mass; EAT: Epicardial Adipose Tissue.

Additional file 2-5. GWI and Different Clinical Parameters (Adjusted for WHR)

	β	SE	95%CI	P
MUH	-240.142	60.258	(-359.046, -121.238)	<0.001
Female	166.426	72.811	(22.754, 310.099)	0.023
Age	9.663	2.811	(4.116, 15.211)	0.001
WHR	-585.315	471.751	(-1516.189, 345.560)	0.216
eGFR	1.773	2.258	(-2.682, 6.229)	0.433
HbA1c	-51.039	19.666	(-89.844, -12.233)	0.01
NT-proBNP	-0.418	0.619	(-1.640, 0.804)	0.501
LVM	1.853	0.829	(0.217, 3.490)	0.027
Never smoking	23.703	68.539	(-111.540, 158.946)	0.73
Drinking	-43.143	90.78	(-222.273, 135.987)	0.635
EAT volume	-1.736	1.921	(-5.527, 2.056)	0.368

Abbreviations: GWI: Global Work Index; MUH: Metabolically Unhealthy; WHR: Waist-Hip Ratio; eGFR: Estimated Glomerular Filtration Rate; HbA1c: Glycated Hemoglobin; NT-proBNP: N-terminal pro–B-type Natriuretic Peptide; LVM: Left Ventricular Mass; EAT: Epicardial Adipose Tissue.
