# Supplementary material for: Analysis of myocardial work in different obesity phenotypes and its association with epicardial adipose tissue
Source: BMC Cardiovasc Disord. 2026 Jul 22;26:936. doi: 10.1186/s12872-026-06298-3 (PMC13679159; doi:10.1186/s12872-026-06298-3)
Supplement: Supplementary file 3 [file 12872_2026_6298_MOESM3_ESM.pdf]

Additional file 3. Modulatory Effect of EAT Volume on MUH and Myocardial Work Parameters

	GWE	GWW	GCW	GWI
**Main Effect**				
MUH	-2.97 (0.43) **	47.3 (10.8) **	-233 (61.4) **	-240 (60.6) **
EAT volume	-0.02 (0.01)	-0.12 (0.35)	-2.66 (1.97)	-1.73 (1.95)
**Modulatory Effect**				
MUH × EAT volume	-0.06 (0.03) **	1.29 (0.64) *	3.28 (3.69)	-2.88 (3.64)
**P for Interaction**	0.02 **	0.046*	0.38	0.43

Covariates including age, sex, Waist-Hip Ratio, smoking history, alcohol consumption history, glycated hemoglobin, N-terminal pro–B-type natriuretic peptide, estimated glomerular filtration rate, and left ventricular mass were included in the regression model. EAT: Epicardial Adipose Tissue; MUH: Metabolically Unhealthy; MW: Global Myocardial Work; P for Interaction: Significance of the modulatory effect; Significance markers: *: $P < 0.05$, **: $P < 0.01$.
