# Supplementary material for: Analysis of myocardial work in different obesity phenotypes and its association with epicardial adipose tissue
Source: BMC Cardiovasc Disord. 2026 Jul 22;26:936. doi: 10.1186/s12872-026-06298-3 (PMC13679159; doi:10.1186/s12872-026-06298-3)
Supplement: Supplementary file 4 [file 12872_2026_6298_MOESM4_ESM.pdf]

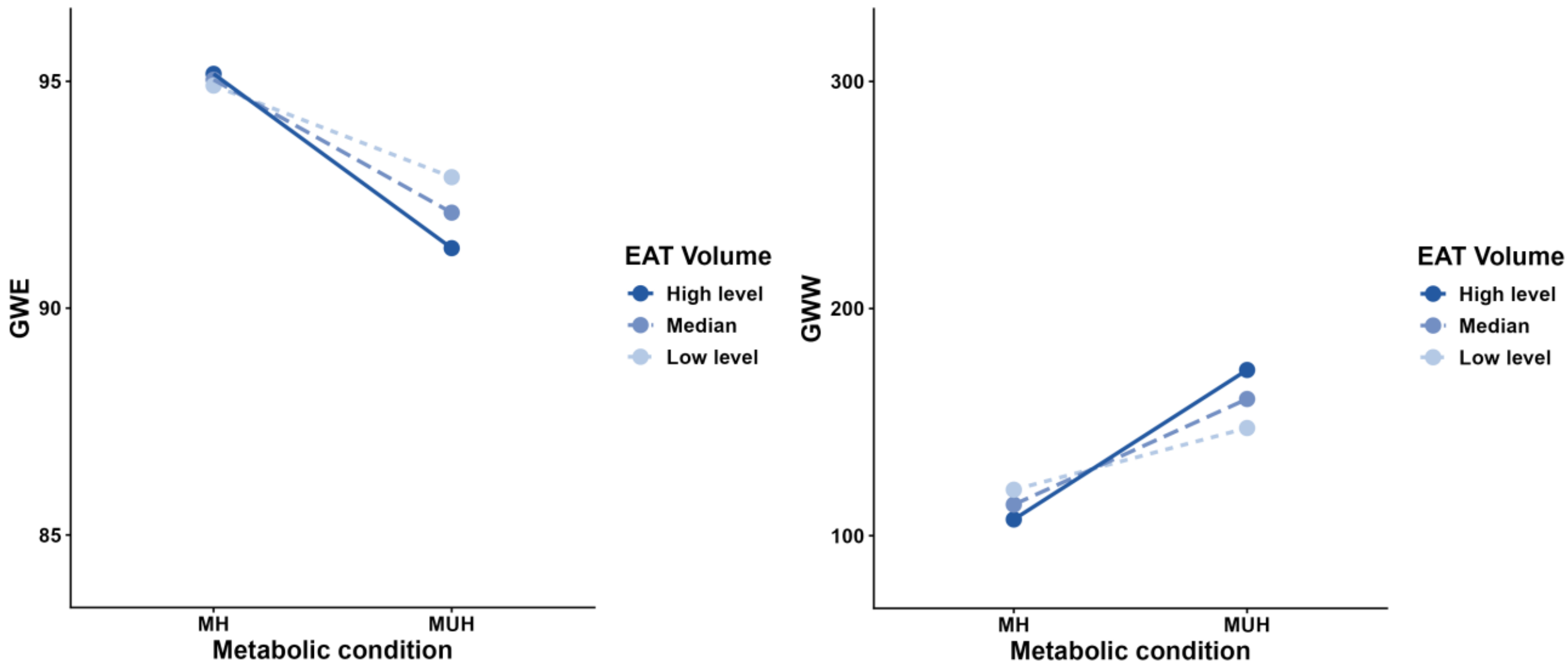

Additional file 4. Moderation effects of GWE and GWW (Adjusted for WHR)
